# A quantitative survey measure of moral evaluations of patient substance misuse among health professionals in California, urban France, and urban China

**DOI:** 10.1186/s13010-023-00148-2

**Published:** 2023-12-05

**Authors:** Anna Yu Lee, Curtis Lehmann, Pengchong Zhou, Bin Xie, Kim D. Reynolds, Alan W. Stacy

**Affiliations:** 1 School of Community & Global Health, Claremont Graduate University, 150 E. 10th St, Claremont, CA 91711 USA; 2 Department of Psychology, Azusa Pacific University, 901 E. Alosta Ave., Azusa, CA 91702 USA; 3 Department of Public Health, California State University Los Angeles, 5151 State University Drive, Los Angeles, CA 90032 USA

**Keywords:** Bioethics, Moral psychology, Multicultural psychology, Substance use disorders, Nursing ethics

## Abstract

**Background:**

The merits and drawbacks of moral relevance models of addiction have predominantly been discussed theoretically, without empirical evidence of these potential effects. This study develops and evaluates a novel survey measure for assessing moral evaluations of patient substance misuse (ME-PSM).

**Methods:**

This measure was tested on 524 health professionals (i.e., physicians, nurses, and other health professionals) in California (*n* = 173), urban France (*n* = 102), and urban China (*n* = 249). Demographic factors associated with ME-PSM were investigated using analyses of variance (ANOVAs) and *t*-tests, with results suggesting that ME-PSM is higher among younger health professionals, nurses (when compared with physicians and other health professionals), and Chinese health professionals (when compared with French and American health professionals).

**Results:**

Results provide preliminary support for the psychometric quality of the survey measure introduced in this study, including the existence of a single latent structure and partial invariance of collected data across countries.

**Conclusion:**

The survey measure for ME-PSM which was developed and tested in the current study appears to hold potential utility for use as a measure of moral views of patient substance misuse. With development, this measure may be used to examine moral evaluations, both as factors of stigma and of other clinical factors associated with the treatment of patients with substance use disorders.

**Supplementary Information:**

The online version contains supplementary material available at 10.1186/s13010-023-00148-2.

## Background

### The importance of moral evaluations of patient behavior

Many debates in the fields of public health, public policy, and bioethics focus on questions about whether specific health behaviors should ever be framed or treated as moral issues. Studies of moral psychology have already explored moral motives [1], moral judgement [2], and moral foundations [3, 4], but there is a specific need for research on moral evaluations in health care, particularly as they relate to clinician well-being, healthcare relationships, and patient outcomes [5]. One central difficulty for this research is the lack of a psychometrically valid measure which assesses these moral framings among health professionals who work with patients with stigmatized disorders. This study introduces the theoretical construct of moral evaluations of patient substance misuse. It is hypothesized that this construct is associated with a wide range of health outcomes (including to whom and how care is provided), as well as with the mechanisms by which these outcomes are effected.

### Moral evaluations of substance misuse

Since a study of moral evaluations of patient behaviors requires empirical specificity [6], this study focuses on moral evaluations specific to substance misuse, a category of health behaviors widely addressed in the literature on stigma and clinical burnout. Those with substance use disorders, especially involving “harder” substances, are stigmatized by the general population [7–10].

When healthcare workers interact with people engaged in behaviors perceived as immoral, these experiences may lead to moral injury.^1^ Research shows that moral injury is associated with ‘burnout’ [12], a condition of career-related physical/psychological exhaustion which many healthcare workers are vulnerable to [13–15]. Although there are numerous factors which contribute to burnout among healthcare workers, the contribution of negative moral evaluations of substance misuse deserves exploration, especially among healthcare professionals who frequently work with people with substance use disorders.

According to moral-relevance models, addiction is at least partially a characterological/spiritual weakness associated with free will and reflective of personal transgressions or spiritual indebtedness [16, 17]. These models provide a foundation for supportive approaches such as 12-step programs, in which participants are taught to seek recovery through a process that includes taking a moral inventory of their lives [18]. These models also provide support for harsher drug laws and stricter public policies pertaining to the management of addictive disorders. Perspectives which deviate from the moral-relevance models argue that in order to avoid devolution into unresolvable debates - as well as to avoid systemic mistreatment of patients with addictive disorders [19, 20]- addiction should never be framed as a moral condition, but rather as a ‘maladaptive coping strategy’, ‘brain disorder’, symptom of ‘sociocultural disadvantage’, ‘learning disorder’, ‘phenomenon of habit,’ or as some form of combination of these [21–24]). Support for these perspectives rests in the notion that the labeling of addictive disorders as ‘moral’ conditions inevitably gives rise to discrimination and stigmatization [25, 26]). Indeed, when clinicians are overly moralistic their perceptions of patients’ conditions become distorted and their relationships with colleagues are adversely affected [27].

Conflicts over ethics may lead to distress and burnout among clinicians, interference with clinician teamwork, and the erosion of trust between patients and practitioners [28]. Additionally, moral evaluations can impede advances in empirical science [29], equitable patient care, workplace professionalism [30, 31] and rapport within patient-practitioner alliances [5]. Nevertheless, moral-relevance models of addiction persist, suggesting an undeniable moral relevance to human habits and behaviors. These models exist in conjunction with models of evolution, neurochemistry, upbringing, and culture [32]; they are not mutually exclusive.

### The need for a measure of moral evaluations of patient substance misuse

Criticisms of moral-relevance views have been advanced largely based upon logical inferences that the stereotypes of weakness, lack of willpower, poor character, and blameworthy necessarily result in stigma and discrimination [33]. Although these arguments are coherent and consistent, there is a lack of empirical evidence of the accuracy of such criticisms. The primary factor for the lack of empirical evidence on this topic has been the lack of a valid and reliable assessment tool.

This study developed and psychometrically evaluated a new measure because - to the extent of this author’s knowledge - no updated, comprehensive, and psychometrically rigorous quantitative survey measure for this construct exists. In developing this measure, moral evaluations were operationalized as the tendency to place behaviors on a continuum of morality and immorality [34] (see Fishbein & Ajzen, 2010). The measure used for this construct draws from the non-moralism subscale of the Substance Abuse Attitude Survey (SAAS), but differs from it in that it focuses on aspects of moral evaluations hypothesized to be relevant to clinical interactions, rather than perceptions regarding drug danger or drug laws.

Such a measure is believed to be useful for various types of research investigations. A measure of moral evaluations could be utilized to determine whether moral-relevance models of substance misuse are associated with stereotypes, prejudice, discrimination, internalized stigma, treatment seeking, and empowerment. Given that the effect of moral evaluations on these outcomes could plausibly interact with other dimensions, including personality (e.g., agreeableness), studies could additionally explore variables that moderate these relationships. The findings from such studies could guide interventions to reduce stigma towards substance misuse. Moreover, studies could evaluate the underlying determinants of moral evaluations, including culture, religiousness, personal experiences, and other relevant variables.

### Overview of the current study

The current study was aimed at providing a measure of assessment of moral evaluations of patient substance misuse by health professionals in different countries and cultures. To this end, a novel measure of moral evaluations of patient substance misuse (ME-PSM) was developed and its psychometric properties were evaluated among health professionals in three culturally-distinct regions of the world: California, urban France, and urban China. To minimize regional biases and increase cross-cultural applicability of the results, the construction of this measure was based on cross-national discussions. In order to examine the factor structure of this measure, confirmatory factor analysis was used to evaluate the psychometric quality of the measure developed in this study. Finally, one-way analyses of variance (ANOVAs) were used to examine demographic and regional factors associated with scores on the ME-PSM.

## Methods

### Participants

All procedures in this study were performed in accordance with the ethical standards of the Institutional Review Board at [blinded] ([blinded] #3490). Recruitment began with the collection of contact information for health professionals (physicians, nurses, and other health professionals) from the websites of major medical schools, nursing schools, schools of psychology, and professional health organizations. Recruitment efforts were limited to post-graduate health programs or hospitals with offices located in California, urban France, and urban China. Potential participants in the California sample were contacted using email, social media posts, and the telephone, with all methods employing use of the same recruitment script. Potential participants in France and China were recruited by email and in person, via intercepts (i.e., a survey approach in which a potential participant is approached by a survey distributor with a recruitment flyer) at two public hospitals in Paris and a combined five public and private hospitals in Shanghai. Recruitment flyers included information on the purpose of the research, the approximate duration of survey participation, and the incentive for survey completion: a raffle entry for $100 (in California), €90 (in France), and RMB ¥700 (in China). All recruitment efforts included wording on approval of study protocol by [blinded] Institutional Review Board (IRB).

Because of deviations from study protocol which occurred during the distribution of paper surveys in France, the paper responses from surveys completed there were discarded. The final sample included physicians, nurses, and ‘other health professionals’ with work experience in California (*n* = 173), urban France (*n* = 102), and urban China (*n* = 249). The pooled sample included participants within the age groups of 18-24 (9.2%), 25-44 (57.2%), and 45+ (33.6%) and included more females (70.2%) than males (29.6%). The sample included physicians (37.0%, nurses (32.6%), and other health professionals (30.3%).

### Data collection

Data for the California sample was collected between July 20 and November 1, 2019, with an online version of the survey delivered via the web platform Qualtrics. Surveys were translated and subsequently proofread by two separate professional translators per non-English language. Data for the French sample was collected between July 1, 2019 and January 6, 2020 using both the online and paper versions of the survey, on which all questions were identical. Finally, data for the Chinese sample was collected between November 1, 2019 and January 6, 2020 using both the online and paper versions of the survey. All participants provided informed consent prior to being given access to the survey. Participants who had been contacted online completed surveys on mobile or desktop devices using a link to the Qualtrics survey; participants who had been recruited in person were given the option of completing the survey on paper or on a mobile device by scanning a QR code that was provided on the study flyer. In the hospitals and health clinics in China, data collection was aided by hospital administrators and clinicians who assisted with flyer distribution. Survey items and the CFA syntax are provided in Additional file 1: Appendix A, and study data is available on the Open Science Framework.

### Measure

The hypothesized construct of moral evaluations of patient substance misuse refers to the tendency to view patient behaviors within a moral framework (i.e., as carrying moral meaning or being situated on a spectrum from moral to amoral). In this study, a measure was developed to assess this construct in relation to substance misuse. The survey questions were adapted from the non-moralism subscale of the Substance Abuse Attitude Survey (SAAS) [35], a measure which has demonstrated reliability and validity [36, 37].

This measure includes six items, scored from 1 to 4, with higher scores indicating evaluations of substance misuse/misusers as more immoral. To provide an example, one survey item included the following prompt and response options: Prompt - “People who use alcohol or other drugs are immoral”; Response Options – “Disagree (1), Somewhat disagree (2), Somewhat agree (3), Agree (4).” A full list of demographic items and survey questions are provided in the Additional file 1. The measure introduced here uses questions regarding free will and cognitive control in relation to substance misuse, to assess one hypothesized factor of moralized views - as opposed to two hypothesized factors (‘drug danger’ and ‘restrictive treatment’), as done in the SAAS. Since the SAAS was developed based on interviews conducted between 1975 and 1985, wording taken from it was updated to exclude outdated terms, such as “street pushers,” and any religiously or denominationally-specific terms, such as “clergymen.” The response options were adapted from wording used for the Pew Research Center’s 2013 Global Attitudes Survey [38].

In order for the questions in the present survey to assess personal perspectives as opposed to normative ethics, they were preceded by instructions which asked participants to respond according to their “personal views,” rather than what they thought they should say based on societal or workplace ethics. Furthermore, so that the questions would examine perceptions of substance *misuse* as opposed to occasional or recreational use, they were preceded by instructions which asked participants to focus on substances/drugs which lead people to seek psychiatric, psychological, or other forms of medical treatment.

### Data analysis

Three single-group confirmatory factor analyses (CFAs) were run in Mplus 7 to evaluate whether the latent construct of ME-PSM satisfied the hypothesized assumption of unidimensionality in each country. Three multiple-group CFAs were run to evaluate hypothesized weak measurement invariance across countries. A mean score of all six ME-PSM items was used to test for differences on ME-PSM by country, age, sex, and occupation. Data fulfilled the requirements of general normality (with a slight positive skew), lack of outliers, and roughly equal sample sizes. Results of Levene’s tests indicated that the assumption of homogeneity of group variances was not met for groupings by geographic region, age range, or occupation, but was met for grouping by gender. To control for Type 1 error rates associated with differences in group variances, post-hoc pairwise group comparisons were run using the Welch’s *t-*test. Data were missing on all ME-PSM items for 0, 4.42, and 7.84% of the California, urban China, and urban France samples, respectively. No participants showed partial data missingness on ME-PSM items. Differences in missing data levels are attributable to variations in survey administration by country; while the California surveys were only available in an online format in which the forced response option was activated, the surveys in France and China were available in both online and paper formats, with no such option activated. Missing data in the CFAs was treated using the default option in Mplus *7* of full information maximum likelihood (FIML). Missing data in the ANOVAs and Welch’s *t*-tests were treated using the default option in *SPSS 25* of pairwise deletion. Multiple perspectives indicate comparative strengths of FIML and pairwise deletion for dealing with missing data over alternate approaches including listwise deletion and multiple imputation [39–41]. The adequacy of the sample size in this study is supported by simulation study which indicated that a six-indicator model with loadings of 0.50 requires a sample size of 90, in order to achieve power of 0.80 [42]. The original measure used in this study included eight items; after CFA and model re-specification to exclude items that loaded on latent factors below 0.50 in any group, the measure retained six items.^2^

Power analyses for ANOVAs and Welch’s *t*-tests were run in G*power. For variables with three or more categories (i.e., age ranges, occupation, and geographic region), post-hoc one-way ANOVA power analyses were conducted; these indicated that with a total sample size of 524 (with three groups), a medium effect size (*f* = 0.25), and an *α* = 0.05, the tests had a power > 0.99. In order to assess the power of tests for variables with only two categories (e.g., gender), a two-tailed biserial correlation power analysis for a medium effect (*ρ =* 0.3), using *α* = 0.05 and power = 0.80, indicated a necessary sample size of 82 per group.^3^ Goodness of model fits were examined using the following model fit criterion: good fit = RMSEA < 0.06, CFI > 0.95; SRMR ≤0.08; acceptable fit = RMSEA ≤ .06; and CFI 0.90-0.95 [44].

## Results

### Psychometric properties

**Fig. 1:**
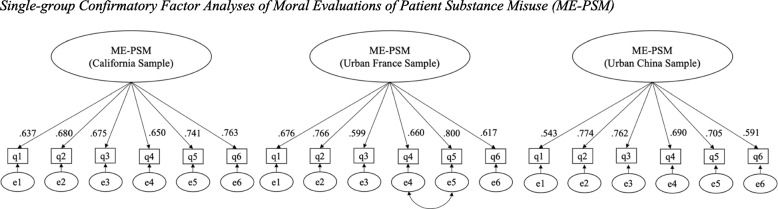
Single-group Confirmatory Factor Analyses of Moral Evaluations of Patient Substance Misuse (ME-PSM). Note. This figure represents the extent to which the items created to measure the hypothesized construct of ME-PSM were consistent with the authors’ understanding of ME-PSM. Factor loadings indicate the explanation of at least 25% of the variance of each survey item by the hypothesized construct of ME-PSM. The bidirectional arrow between items 4 and 5 in the France group indicate manual correlation of items 4 and 5 to account for detected correlation of residuals on these items. All factor loadings were found to be significant at *p* < .01. Estimates were standardized

Factor loadings indicate that at least 25% of the variance of each item is explained by its hypothesized factor of ME-PSM (see Fig. 1). In Mplus, modification indices^4^ above 10 are displayed, in order to flag possible instances of local dependence (i.e. situations which occur when the residuals of two or more items covary (i.e. are correlated) after adjusting for a latent factor [47], which is a problem in that it potentially introduces bias to estimations [48]. In the France group, question items 4 and 5 were flagged. To correct for this, these items were set to be correlated with one another. The addition of one parameter in the France group (i.e., Item 4 with Item 5) did not substantially alter the factor loadings in the France CFA, and likely arose because of overlapping wording between indicator pairs.

### Measurement invariance

In order to determine the functioning of ME-PSM across countries, measurement invariance was evaluated. This was done through a process of comparing increasingly restricted models and retaining the most parsimonious model that demonstrated adequate model fit [49–51]. Model 1 tested for invariance at the weakest level, sometimes referred to as the level of ‘configural invariance,’ in which the same pattern of fixed and free factor loadings is specified for each group. This least-restricted model included no equality constraints across groups. Model 2 tested for invariance at a stronger level, sometimes referred to as the level of ‘metric invariance, in which factor loadings for similar items are tested for invariance across groups [52]. In this model, equality constraints were added to all factor loadings across groups (except for those set to 1.0, to establish the scale of measurement). If the use of Model 2 was supported, an increasingly restricted Model 3 would be run and compared against Model 2.

Differences between the increasingly nested models were tested using the Satorra-Bentler χ^2^ correction formula for robust parameter estimation [49, 53]. The χ^2^ difference between Model 1 and Model 2 was significant at *p* < 0.01, suggesting retention of Model 1 (the less parsimonious model with more parameters) and establishment of measurement invariance at the weakest (i.e., the “configural”) level. While configural invariance implies that results across groups can be considered at least on a conceptual level, analyses must be tempered with the recognition that constructs are measured somewhat differently across groups [49–52] (Table 1).

**Table 1:** Fit Indices of confirmatory factor analyses for moral evaluations of patients’ substance misuse

	AIC	SRMR	df	CFI	RMSEA	χ^2^	*p*-values
Multiple-group Model 1: No equality constraints specified							
	7233.781	.029	26	.991	.037	32.072	.1907
Multiple-group Model 2: Equality constraints inserted on factor loadings only							
	7263.333	.085	35	.949	.074	68.927	.000
Single-group (California)							
	2208.069	.031	9	1.00	.000	8.615	.4736
Single-group (urban France)							
	1174.954	.030	8	.980	.062	11.173	.0033
Single-group (urban China)							
	3850.758	.027	9	.988	.043	13.237	.0001

### Descriptive analysis

Results from Welch’s t-tests run in *SPSS 25* are provided in Table 2. Significant differences on the mean scores of ME-PSM are seen between urban China and California (*t* = − 13.014, *p* < 0.01) and between urban China and urban France (*t* = − 11.027, *p* < 0.01), but not between California and urban France (*t* = .110, *p* = 0.912); mean scores are highest in urban China when compared to California and urban France. Significant differences on the ME-PSM mean scores across countries are shown between participants aged 25 to 44 and those over 45 (*t* = 3.94, *p* < 0.01) and between participants aged 18 to 24 and over 45 (*t* = 4.57, *p* < 0.01), but not between participants aged 18 to 24 and 25 to 44 (*t* = 1.61, *p* = 0.109), with mean scores being lowest among participants over 45 years old when compared with participants aged 18 to 24 and 25 to 44. Significant differences on the ME-PSM mean scores across countries are seen between physicians and nurses (*t* = − 3.46, *p* < 0.01), between nurses and ‘other health professionals’ (*t* = 5.15, *p* < 0.01), and between physicians and other health professionals (*t* = 2.05, *p* = 0.04); mean scores are highest among nurses, followed by physicians, then by other health professionals. No significant differences on the ME-PSM mean scores are found between males and females (*t* = .379, *p* = .554).

**Table 2:** Means, standard deviations, and welch’s t-tests of moral evaluations of patient’ substance misuse

	California (a)		Urban France (b)		Urban China (c)			
	*n*	*M* (*SD*)	*n*	*M* (*SD*)	*n*	*M* (*SD*)	Group *M*	*t* tests
Overall	173	1.59(.63)	102	1.58(.57)	249	2.51 (0.76)	–	a-b, *p* = .912 b-c, *p* < .01 a-c, *p* < .01
*Age Group*								
18-24 (d)	7	1.93(.53)	3	1.55(.10)	38	2.45(.67)	2.32	d-e, *p* = .109
25-44 (e)	80	1.58(.66)	58	1.65(.62)	162	2.54(.80)	2.11	e-f, *p* < .01
45+ (f)	86	1.58(.60)	41	1.49(.52)	49	2.45(.71)	1.80	d-f, *p* < .01
*Gender*								
Male (g)	34	1.71(.69)	54	1.57(.60)	67	2.48(.74)	1.99	g-h, *p* = .554
Female (h)	138	1.57(.61)	48	1.59(.54)	182	2.52(.77)	2.04	
*Occupation*								
Physician (i)	42	1.61(.67)	65	1.56(.55)	87	2.46(.67)	1.98	i-j, *p* < .01
Nurse (j)	46	1.64(.59)	17	1.61(.11)	108	2.65(.82)	2.28	j-k, *p* < .01
Other HP (k)	85	1.56(.64)	20	1.63(.68)	54	2.28(.72)	1.81	i-k, *p* = .04

ME-PSM items scale range: 1–4

## Discussion

### Weak measurement invariance

In this study, measurement invariance was only established at the configural (i.e., weak) level by country, suggesting that the instruments used here operate differently in each country. This finding is similar to that of Iurino and Saucier (2020), who found that the Moral Foundations Questionnaire [54] did not converge well across 27 countries in areas including North America, Western Europe, and East Asia. Culturally specific interpretations of wording of survey items may also have been associated with apparent difference by country. Research has shown that different cultures hold differing beliefs about what is moral, as shaped by individual and/or collective worldviews [55], sanctity-of-life vs. quality-of-life ethics [56], or liberal/social, welfarist/deontological views [57].

Another cause of weak measurement invariance may have been differences in the survey administration approaches between countries (i.e., surveys were administered online-only in California, and either online or on-paper in urban France and urban China). Finally, differences may have been associated with differences in the occurrence of social desirability bias by country. To explain – the concept of ‘moral evaluations’ might be viewed or valued differently by country, and health professionals from given countries might respond in manners biased by their feelings about how they should respond to questions about moral evaluations in clinical settings.

### Group differences

While configural invariance would certainly imply that group differences merit discussion, weak invariance indicates caution in interpreting group differences, which must be considered preliminary. One finding on group differences was that ME-PSM was highest in China. Mean scores on ME-PSM were largely higher in urban China when compared with California (Cohen’s *d* = 1.39) and urban France (Cohen’s *d* = 1.32). This finding may be studied through an exploration of the values that shape each country’s moral landscape. Differences in moral foundations between countries were briefly discussed in the Introduction. In additional analyses not discussed here, both religiosity and authoritarianism were associated with ME-PSM in the California sample with the effect being stronger between religiosity and ME-PSM, when compared with the effect between authoritarianism and ME-PSM. Data on religiosity and authoritarianism were not collected in France or China, but future studies might explore the effects of these factors and others in China, in order to understand why ME-PSM is higher in that country. Given the relatively secular nature of urban China when compared with the United States, however, it appears that certain factors must be even more influential than religiosity when it comes to shaping concepts regarding the moral nature of SUDs. Given traditions of collectivism in China, such concepts in the Chinese context might be shaped by thoughts about how SUDs impact the social lives and communities of patients affected by them. Concepts of ME-PSM in China may also relate to the culturally specific notion of *losing face* - the moral-emotional state of losing respect among one’s peers and social circle. In China, mental illness is often associated with a loss of face or sense of stigma, in ways that bring shame not only to the mentally ill, but also to their family members [58]. Because of the effects of mental illness on the reputational status of a patient’s family and social network, it is possible that the high levels of ME-PSM reported in China reflect a greater consideration of the effects of substance misuse on family members and social groups, as opposed to the effects of substance misuse on just the patients themselves.

The finding that ME-PSM was largely lower among participants over 45 years old, when compared with participants aged 18 to 24 (Cohen’s *d* = 0.73) and moderately lower when compared with participants aged 25 to 44 (Cohen’s *d* = 0.38), provides avenues for speculation. This finding may indicate that as people age, any views that they might have had about substance misuse being amoral become less extreme. This change in perspective may be a result of what people witness or personally experience regarding drug use over the course of the first 45 (or more) years of their life. This difference may also represent a generational difference in perspective; it is possible that participants in the over-45 age group belong to generational cohorts in which drug use is more common or accepted.

ME-PSM was also found to be highest among nurses, when compared with physicians (Cohen’s *d* = 0.36) and ‘other health professionals’ (Cohen’s *d* = 0.57). This finding can be interpreted in light of studies which indicated that nurses were less permissive toward substance abuse than social workers [59]. Similarly, nurses were less tolerant and more morally condemning of alcohol and drug misuse, when compared with other health care professionals [60]. Although the studies are several decades old, nurses recommended more punitive responses to problem drinking, while physicians, psychologists, and workers advocated for more therapeutic responses [61] and nurses were more likely to recommend compulsory treatment for problem alcohol misuse when compared to physicians [62].

### Limitations

Given logistical constraints of data collection, the sample sizes used in this study were only large enough to detect medium effect sizes. Further, the results of this study were limited by the use of a convenience sample of self-report data. Since people may feel compelled to respond in certain ways (particularly in professional settings) to questions about moral values, the data collected on ME-PSM may have been biased by social desirability and demand characteristics. The generalizability of findings is furthermore limited by the ambiguity of the category of ‘other health professional’, and the fact that this instrument was not studied among health care providers who specifically or exclusively work with patients with SUDs. While this study used a CFA to examine the factor structure of the hypothesized construct of ME-PSM, additional approaches are needed to determine the validity of this measure. Finally, future studies might examine complex psychometric properties associated with the instrument introduced in this study.

## Conclusion

Moral evaluations represent an important concept that has social validity, or the characteristic of being socially important, given the ongoing tendency of the public to evaluate substance misuse as an immoral behavior. This study introduces a measure of moral evaluations that could be useful in studying the nature of moral-relevance models. With further development, the measure in this study could be used to examine potential associations between moral evaluations and stigma/discrimination against patients who misuse substances.

Even if studies find a positive association between increased moral evaluations of substance misuse and discrimination, it is critical for researchers to appreciate that there may be variables that moderate this relationship. There are numerous variables that may be relevant to such a relationship. One concept could be termed moral prognosis which could involve a continuum that, on one extreme, sees moral character as fixed and unchanging and, on the other extreme, believes that people are capable of profound moral transformation. A health professional who evaluated substance morally but held an optimistic moral prognosis would potentially be optimistic about the patient’s likelihood of recovery and could provide treatment absent of discrimination, particularly if the health professional believed that moral transformation could be facilitated through appropriate clinical care.

Another variable could include moral compassion, which could involve the tendency to perceive those who are perceived to have poor moral character as deserving of compassionate treatment and concern. These variables could have implications for the relationship between moral evaluations and discrimination.

The health professional’s moral evaluations are an important consideration for treatment of patients. A straightforward explanation might assume that moral evaluations of patient substance misuse are directly and inversely related to patient care. This outcome is feasible if the health professional were to give up on the patient, develop a sense of impatience with or disliking for the patient, or feel discouraged or demoralized in their role as a clinician. Yet, the effect of moral evaluations on treatment of patients can potentially be moderated by other factors. Moral evaluations could coincide with activation of a sense of moral duty to support the patient, the triggering of a consideration of psychological, sociocultural, or environmental factors which the patient may be experiencing, and an increased likelihood to suggest treatment approaches which might support the patients’ inner life. This study provided a psychometrically evaluated scale that could be useful for future research on this topic, including identifying the mechanisms which drive health professionals toward various responses to patient behaviors.

While the instrument developed in this study operated differently by country, results nevertheless provide some preliminary evidence of the utility of this survey measure in assessing the unidimensional construct of ME-PSM in different cultural settings among health professionals. Future research could validate the ME-PSM survey among other populations for whom moral judgements may be of clinical or social relevance (e.g., patients, patients’ partners, police officers, corrections officers, etc.). Future research may also explore intrapersonal and interpersonal predictors and outcomes of ME-PSM pertaining to help-seeking behaviors, adherence to treatment recommendations, recovery rates, practitioner-patient rapport, and may explore moral evaluations of a wider range of patient behaviors (including diet, exercise, and risky sexual behaviors, among others).

### Supplementary Information

**Additional file 1: Appendix A.** Survey. **Appendix B.** Syntax for Measurement Invariance (Model 1). **Appendix C.** Syntax for Measurement Invariance (Model 2). **Appendix D.** Syntax for Single-Group CFAs

## Data Availability

All study data is available on the Open Science Framework.

## Abbreviations

ME-PSM: Moral Evaluation of Patient Substance Misuse
ANOVAs: Analyses of Variance
SUDs: Substance Use Disorders
SAAS: Substance Abuse Attitude Survey
RMB: Ren Men Bi (Chinese Currency)
QR code: Quick Response code
CFA: Confirmatory Factor Analysis
FIML: Full Information Maximum Likelihood
SPSS: Statistical Package for Social Sciences
RMSEA: Root Mean Square Error of Approximation
CFI: Comparative Fit Index
SRMR: Standardized Root Mean Square Residual
AIC: Akaike Information Criterion
Df: Degrees of Freedom

## Footnotes

1 Moral injury has been defined as an experience that includes involvement with acts that violate a person’s deeply held moral beliefs [11].

2 The minimum loading of 0.5 was selected based on the recommendations of a reference text on SEM [43].

3 Results of G*power analysis run for small effect sizes: 1.) A post-hoc one-way ANOVA power analyses indicated that with a total sample size of 524 (with three groups), a medium effect size (*f* = 0.1), and an *α* = 0.05, a test had a power = .521. 2.) A power analysis for detecting a small two-tailed biserial correlation between two groups (*ρ =* 0.1) using *α* = 0.05 and power = 0.80 indicated a necessary sample size of 779 per group.

4 A modification index (i.e. a univariate Lagrange Multiplier) is expressed as chi-square statistics with one degree of freedom. It is individually calculated for every zero-fixed path, and approximates the amount by which chi-square would decrease (and the fit of the model would increase) if a certain path was added to the model by freely estimating one of the fixed-to-zero paths [45, 46].
