# Supplementary material for: A quantitative survey measure of moral evaluations of patient substance misuse among health professionals in California, urban France, and urban China
Source: Philos Ethics Humanit Med. 2023 Dec 5;18:18. doi: 10.1186/s13010-023-00148-2 (PMC10696895; doi:10.1186/s13010-023-00148-2)
Supplement: Supplementary file 1 — Additional file 1: Appendix A. Survey. Appendix B. Syntax for Measurement Invariance (Model 1). Appendix C. Syntax for Measurement Invariance (Model 2). Appendix D. Syntax for Single-Group CFAs [file 13010_2023_148_MOESM1_ESM.docx]

Appendix A

Survey

*(English)*

| Age | |
| --- | --- |
| Question  What age category are you in? | Response options   - 18-24 (1) - 25-44 (2) - 45+ (3) |
| Sex | |
| Question  What is your gender? | Response options   - Male = (1) - Female = (2) - Other = (3) |
| Occupation | |
| Question  Are you a health professional who currently or previously  worked in California?  What is your primary occupation? | Response options   - Yes (1) - No (2) - Physician (1) - Nurse (2) - Other (3) |
| Moral evaluation of patient behavior specific to substance misuse (ME-PSM)  *Instructions: Please answer the following questions only according to your personal views (as opposed to the rules you might be taught in society or at work). In answering these questions, please consider ‘substances’ and ‘drugs’ that lead people to seek psychiatric, psychological, or other medical treatment. Please remember that all responses to this survey are confidential.* | |
| Questions   1. Substance use is associated with a weak will. 2. People who use alcohol or other drugs are immoral. 3. The decision to use alcohol or other drugs is a moral decision 4. People who use alcohol or other drugs should think about the morality of their actions. 5. Moral people avoid the use of alcohol or other drugs. 6. Substance use is a matter of right and wrong. | Response options   - Disagree (1) - Somewhat disagree (2) - Somewhat agree (3) - Agree (4) |

*(Français)*

| Âge | |
| --- | --- |
| Question  Dans quelle catégorie d'âge êtes-vous? | Options de réponse   - 18-24 (1) - 25-44 (2) - 45+ (3) |
| Sexe | |
| Question  Quel est ton sexe? | Options de réponse   - Homme (1) - Femme (2) - Autre (3) |
| Titre de votre poste | |
| Question  Quelle est votre occupation principale? | Options de réponse   - Médeci (1) - Infirmière (2) - Autre (3) |
| Évaluation morale du comportement du patient spécifique à la toxicomanie  Veuillez répondre aux questions suivantes uniquement en fonction de vos opinions personnelles (par opposition aux règles qui pourraient vous être enseignées dans la société ou au travail). Pour répondre à ces questions, veuillez considérer les «substances» et les «drogues» qui poussent les gens à rechercher un traitement psychiatrique, psychologique ou autre. N'oubliez pas que toutes les réponses à ce sondage sont confidentielles. | |
| Des Questions   1. La consommation de substances est associée à une volonté faible. 2. Les personnes qui consomment de l'alcool ou d'autres drogues sont immorales. 3. La décision de consommer de l'alcool ou d'autres drogues est une décision morale. 4. Les personnes qui consomment de l'alcool ou d'autres drogues devraient penser à la moralité de leurs actes. 5. Les personnes morales évitent la consommation d'alcool ou d'autres drogues. 6. La consommation de substances psychoactives est une question de bien et de mal. | Options de réponse   - Pas d'accord (1) - Plutôt en désaccord (2) - Plutôt d'accord (3) - D'accord (4) |

*(中文)*

| 年龄 | |
| --- | --- |
| 题  请圈年龄? | 回应选项   - 18-24 (1) - 25-44 (2) - 45+ (3) |
| 性别 | |
| 题  请圈性别? | 回应选项   - 男 (1) - 女 (2) - 其他 (3) |
| 占用 | |
| 题  请圈职称? | 回应选项   - 医生 (1) - 护士 (2) - 其他医疗卫生从业者 (3) |
| 针对(非安全物质使用)的患者行为的道德评估  请仅根据您的个人意见回答以下问题（而不是基于您在社会或工作中所接受的规定或教导). “非安全物质”或“药物”是指频发地导致人们进行精神、心理或其他治疗的物质。此项研究中针对的“物质”是指像酒精，香烟，大麻，海洛因和可卡因这类物质，而并非如咖啡因，糖等可能改变或伤害身心但不太会导致人们寻求医疗治疗的物质。对此调研的所有答复都是保密的 | |
| 问题   1. 物质滥用与意志薄弱有关. 2. 使用酒精或其他药物的人是不道德. 3. 使用酒精或其他药物属于道德决定. 4. 使用酒精或其他药物的人应该考虑他们行为的道德性. 5. 有道德的人会避免使用酒精或其他药物. 6. 物质滥用是一个是非问题. | 回应选项   - 不同意 (1) - 不同意 (2) - 比较同意 (3) - 同意 (4) |

Appendix B

Syntax for Measurement Invariance (Model 1)

Mplus VERSION 7.4

DATA: File is Dissertation Data China France Cali Mplus Input 8.2.20.txt;

VARIABLE:

NAMES ARE

Job Sex Age

MEPSMi1 MEPSMi2 MEPSMi3 MEPSMi4 MEPSMi5 MEPSMi6 MEPSMi7 MEPSMi8

COMPi1 COMPi2 COMPi3 COMPi4 COMPi5

SEi1 SEi2 SEi3 SEi4 SEi5 SEi6 SEi7 SEi8 SEi9 SEi10

OPTi1 OPTi2 OPTi3 OPTi4 OPTi5

MESi1 MESi2 MESi3 MESi4 MESi5 MESi6 MESi7 MESi8 MESi9

Country

Religios1 Religios2 Religios3 Religios4 Religios5

Authori1 Authorit2 Authorit3 Authorit4 Authorit5 Authorit6;

USEVARIABLES ARE

MEPSMi2 MEPSMi3 MEPSMi4 MEPSMi5 MEPSMi6 MEPSMi7 Country;

Grouping IS country (1 = Cali 2 = France 3 = China);

Missing are .;

ANALYSIS:

estimator=MLR;

MODEL:

MEPSMlf by MEPSMi2-MEPSMi7;

[MEPSMlf@0];

MODEL France:

MEPSMlf by MEPSMi3-MEPSMi7;

[MEPSMi2-MEPSMi7];

MEPSMI5 WITH MEPSMI4;

MODEL China:

MEPSMlf by MEPSMi3-MEPSMi7;

[MEPSMi2-MEPSMi7];

output:

SAMPSTAT MODINDICES STAND RESIDUAL;

Appendix C

Syntax for Measurement Invariance (Model 2)

Mplus VERSION 7.4

DATA: File is Dissertation Data China France Cali Mplus Input 8.2.20.txt;

VARIABLE:

NAMES ARE

Job Sex Age

MEPSMi1 MEPSMi2 MEPSMi3 MEPSMi4 MEPSMi5 MEPSMi6 MEPSMi7 MEPSMi8

COMPi1 COMPi2 COMPi3 COMPi4 COMPi5

SEi1 SEi2 SEi3 SEi4 SEi5 SEi6 SEi7 SEi8 SEi9 SEi10

OPTi1 OPTi2 OPTi3 OPTi4 OPTi5

MESi1 MESi2 MESi3 MESi4 MESi5 MESi6 MESi7 MESi8 MESi9

Country

Religios1 Religios2 Religios3 Religios4 Religios5

Authori1 Authorit2 Authorit3 Authorit4 Authorit5 Authorit6;

USEVARIABLES ARE

MEPSMi2 MEPSMi3 MEPSMi4 MEPSMi5 MEPSMi6 MEPSMi7 Country;

Grouping IS country (1 = Cali 2 = France 3 = China);

Missing are .;

ANALYSIS:

estimator=MLR;

MODEL :

MEPSMlf by MEPSMi2(f);

MEPSMlf by MEPSMi3(a);

MEPSMlf by MEPSMi4(b);

MEPSMlf by MEPSMi5(c);

MEPSMlf by MEPSMi6(d);

MEPSMlf by MEPSMi7(e);

MEPSMlf;

[MEPSMi2];

[MEPSMi3];

[MEPSMi4];

[MEPSMi5];

[MEPSMi6];

[MEPSMi7];

[MEPSMlf@0];

MODEL France:

MEPSMlf by MEPSMi2(f);

MEPSMlf by MEPSMi3(a);

MEPSMlf by MEPSMi4(b);

MEPSMlf by MEPSMi5(c);

MEPSMlf by MEPSMi6(d);

MEPSMlf by MEPSMi7(e);

MEPSMlf;

[MEPSMi2];

[MEPSMi3];

[MEPSMi4];

[MEPSMi5];

[MEPSMi6];

[MEPSMi7];

MEPSMI5 WITH MEPSMI4 ;

MODEL China:

MEPSMlf by MEPSMi2(f);

MEPSMlf by MEPSMi3(a);

MEPSMlf by MEPSMi4(b);

MEPSMlf by MEPSMi5(c);

MEPSMlf by MEPSMi6(d);

MEPSMlf by MEPSMi7(e);

MEPSMlf;

[MEPSMi2];

[MEPSMi3];

[MEPSMi4];

[MEPSMi5];

[MEPSMi6];

[MEPSMi7];

output:

SAMPSTAT MODINDICES STAND RESIDUAL;

Appendix D

Syntax for Single-Group CFAs

Mplus VERSION 7.4

DATA: File is Dissertation Data China France Cali Mplus Input 8.2.20.txt;

VARIABLE:

NAMES ARE

Job Sex Age

MEPSMi1 MEPSMi2 MEPSMi3 MEPSMi4 MEPSMi5 MEPSMi6 MEPSMi7 MEPSMi8

COMPi1 COMPi2 COMPi3 COMPi4 COMPi5

SEi1 SEi2 SEi3 SEi4 SEi5 SEi6 SEi7 SEi8 SEi9 SEi10

OPTi1 OPTi2 OPTi3 OPTi4 OPTi5

MESi1 MESi2 MESi3 MESi4 MESi5 MESi6 MESi7 MESi8 MESi9

Country

Religios1 Religios2 Religios3 Religios4 Religios5

Authori1 Authorit2 Authorit3 Authorit4 Authorit5 Authorit6;

USEOBSERVATIONS = country EQ 1; ! or 2 or 3, depending on country

USEVARIABLES ARE

MEPSMi2 MEPSMi3 MEPSMi4 MEPSMi5 MEPSMi6 MEPSMi7 ;

Missing are .;

ANALYSIS:

estimator=MLR;

MODEL:

MEPSMlf by MEPSMi2-MEPSMi7;

output:

SAMPSTAT MODINDICES STAND RESIDUAL;
